# *Mycobacterium avium* subsp. *paratuberculosis* exploits miRNA expression to modulate lipid metabolism and macrophage polarisation pathways during infection

**DOI:** 10.1038/s41598-022-13503-8

**Published:** 2022-06-11

**Authors:** Kathryn Wright, Rachel Mizzi, Karren M. Plain, Auriol C. Purdie, Kumudika de Silva

**Affiliations:** Sydney School of Veterinary Science, The University of Sydney, Faculty of Science, Sydney, NSW Australia

**Keywords:** Innate immunity, miRNAs, Bacterial infection, Infection

## Abstract

Pathogenic mycobacteria including *Mycobacterium avium* subsp. *paratuberculosis* (MAP), the causative agent of Johne’s disease, manipulate host macrophages to persist and cause disease. In mycobacterial infection, highly plastic macrophages, shift between inflammatory M1 and permissive M2 phenotypes which alter the disease outcome and allow bacteria to survive intracellularly. Here we examine the impact of MAP infection on polarised macrophages and how increased lipid availability alters macrophage phenotype and bacterial persistence. Further, we assess if host microRNA (miRNA) are sensitive to macrophage polarisation state and how MAP can drive their expression to overcome innate responses. Using in vitro MAP infection, we find that increasing lipid availability through supplementing culture media with exogenous lipid increases cellular nitric oxide production. Lipid-associated miRs -19a, -129, -24, and -24-3p are differentially expressed following macrophage polarisation and lipid supplementation and are further regulated during MAP infection. Collectively, our results highlight the importance of host lipid metabolism in MAP infection and demonstrate control of miRNA expression by MAP to favour intracellular persistence.

## Introduction

The interaction between macrophages and virulent mycobacteria is a major determining factor in the outcome of infection. Macrophages form one of the first lines of defence against invading pathogens and are often hijacked by pathogenic mycobacteria to provide a survival niche, protected from the host immune system^1^. Macrophages may be categorised into subsets, including either classically activated proinflammatory M1, or alternatively activated anti-inflammatory M2 phenotypes. Macrophage polarisation is an adaptable process and is determined by the type of immune stimuli and pathogen signal^2^. During mycobacterial infection there is, however, a high degree of plasticity in macrophages, often indicative of disease state and stage of infection^3,4^.

*Mycobacterium avium* subsp. *paratuberculosis* (MAP) is a pathogenic mycobacterium which causes the enteric granulomatous disease, Johne’s disease. Following uptake via the faecal oral route, MAP is initially able to cross the intestinal epithelial barrier via specialised microfold or M cells within Peyer’s patches where it is then phagocytosed by macrophages^5^. A hallmark of pathogenic mycobacteria including MAP is the ability to manipulate a range of macrophage functions, including preventing phagosome maturation, lysosomal degradation, and preventing macrophage apoptosis^6–8^. This blockade of innate macrophage defences allow MAP to persist within host macrophages, leading to the formation of granulomatous lesions and long-term chronic infections. Studies on the polarisation of host macrophages during MAP infection have identified mixed populations of both M1 and M2 macrophages rather than a distinct classical or alternative dominance^9^. Further, macrophage polarisation state is largely related to disease state, with clinically infected animals showing lower numbers of microbicidal M1 macrophages, while subclinically infected animals display similar of numbers of M1 and M2 “regulatory” macrophages^10^.

A key metabolic process in both the uptake of mycobacteria into macrophages and their survival inside the macrophage, is lipid and cholesterol metabolism. Accumulation of cholesterol in macrophages infected with mycobacteria is essential for blocking innate macrophage defences, allowing macrophages to become permissive to bacterial survival^11,12^. This pathway is modulated by MAP to dysregulate host lipid metabolism genes and accumulate intracellular cholesterol, leading to the formation of lipid-rich foam cells^13–17^. Post-transcriptional regulation of macrophage responses and lipid metabolism by non-coding RNA such as microRNA (miRNA) may also be regulated by MAP to further ensure growth and survival^18^.

miRNA are small (19–25 nt) single stranded molecules that exert post-transcriptional control over protein translation. Through complementary untranslated (UTR) region binding to target mRNA, miRNAs are able to reduce protein translation by transiently binding to, or degrading bound mRNA^19^. miRNAs are considered master regulators of gene expression and are involved in many biological and immune pathways. These molecules are differentially expressed during MAP infection and have been implicated in macrophage polarisation^20–24^.

Due to the central role of macrophages in MAP infection, and the different cellular immune responses of M1 and M2 macrophage phenotypes, we examined the effect of MAP infection on polarised macrophages. We further supplemented polarised and MAP infected macrophages with lipid to assess the impact on phenotype and host response. We also investigated whether miRNAs are sensitive to macrophage polarisation and if MAP infection can overcome these factors to alter their expression and drive pathogenesis.

## Results

### Murine macrophage polarisation

**Figure 1:**
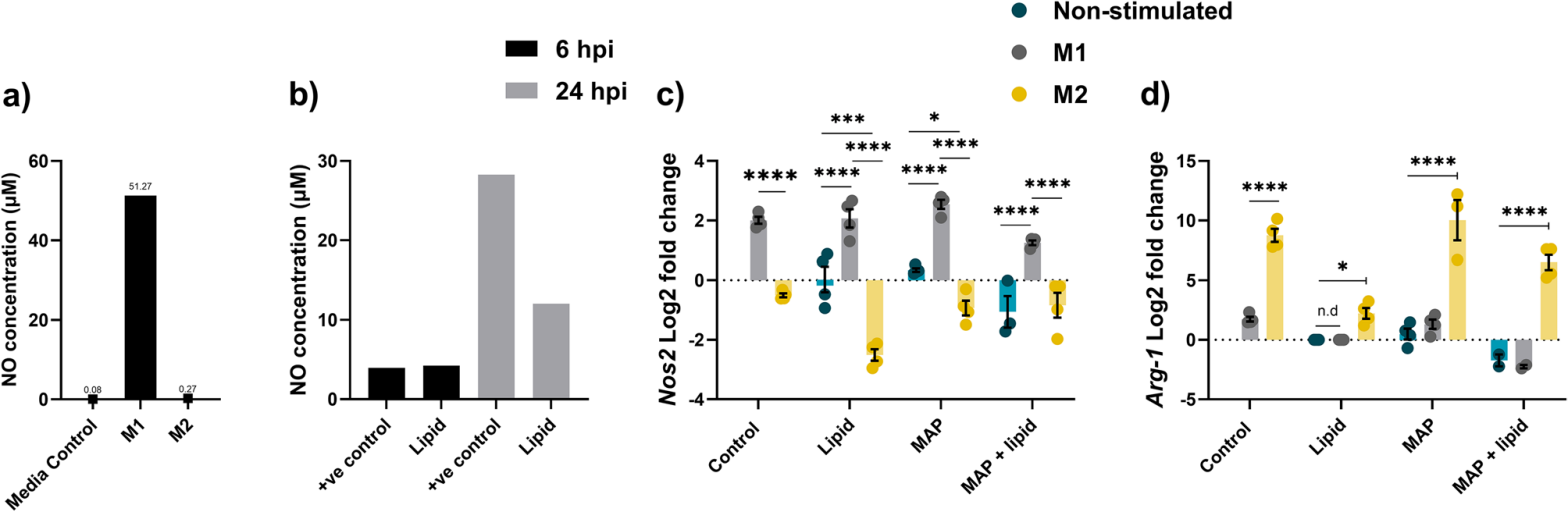
MAP infection maintains an M1 macrophage state during early infection. RAW264.7 macrophages were polarised to an M1 or M2 state and further supplemented with exogenous lipid and/or infected with MAP. Cellular NO production was measured using a Griess assay at (**a**) 22 h post polarisation (n = 3) and (**b**) 6 and 24 h post lipid supplementation (n = 2); LPS stimulation was used as a positive control. Expression of *Nos2* (M1 marker) (**c**) and *Arg-1* (M2 marker) (**d**) was assessed by qPCR at 24 hpi. *Arg-1* was not detected (n.d) in unstimulated and M1 macrophages supplemented with lipid (n = 2). Controls are the polarised cells without lipid or MAP. Error bars show standard deviation. *p ≤ 0.05, **p ≤ 0.01, ***p ≤ 0.001, ****p ≤ 0.0001).

Macrophage differentiation was assessed by gene expression analysing common M1 and M2 markers (*Nos2* and *Arg-1* respectively), as well as cellular nitric oxide (NO) secretion, while MAP infection was confirmed microscopically. As expected, M1 but not M2 polarised macrophages produced high levels of NO (Fig. 1a). To characterise the effect on macrophage phenotype following supplantation with exogenous lipid, unpolarised macrophages were supplemented with lipid and cellular NO production was measured and compared to LPS (positive control). At 6 h, lipid supplementation resulted in an increase in cellular NO production in unstimulated macrophages on par with positive control levels. This increase in cellular NO was also observed at 24 h, indicating that macrophages provided with exogenous lipid are shifted towards an inflammatory M1 phenotype (Fig. 1b).

To further characterise the activation state of polarised macrophages supplemented with lipid and/or infected with MAP, the expression of M1 and M2 marker genes *Nos2* and *Arg-1* was measured. In line with observed NO production, M1 polarised macrophages displayed increased expression of the M1 marker *Nos2* and low expression of M2 marker *Arg-1,* while the combination of lipid and MAP infection did not alter the phenotype or activation state of these cells (Fig. 1c,d). In M1 polarised macrophages, lipid and MAP reinforced the M1 phenotype, however these factors had the opposing effect in M2 differentiated cells, shifting the macrophages away from the M2 phenotype.

**Figure 2:**
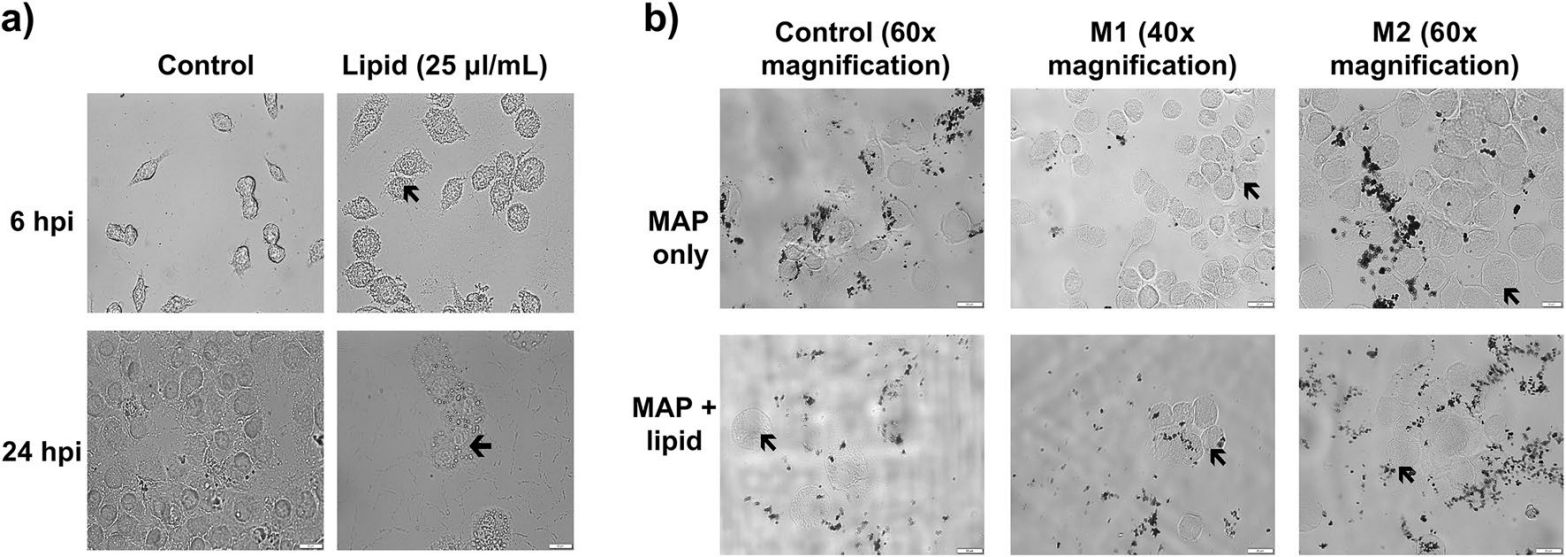
Lipid supplementation aids in the formation of foam cells and contributes to bacterial survival during MAP infection. (**a**) RAW264.7 macrophages were supplemented with exogenous lipid and imaged at 6 and 24 hpi. Lipid droplet formation within cells leading to foam cell formation (indicated by black arrows) were observed at both timepoints in macrophages supplemented with lipid. (**b**) Polarised RAW264.7 macrophages were infected with MAP and further supplemented with lipid prior to imaging at 6 hpi. Cells were heat fixed at 85 °C for 45 min prior to imaging, and all images acquired using an BX61 Olympus microscope. Scale bar represents 20 μm.

Macrophages supplemented with lipid were imaged at 6 and 24 h post infection (hpi) to assess phenotype and foam cell formation. At both 6 and 24 hpi, visible foam cells were observed compared to control macrophages. When lipid supplemented macrophages were infected with MAP, foam cells were still observed at 6 hpi, however bacterial clearance was reduced compared to non-supplemented MAP infected macrophages (Fig. 2). Overall, this suggests that MAP infection and lipid supplementation drive an M1 inflammatory macrophage phenotype in early infection, even in previously M2 polarised macrophages, and the formation of foam cells during MAP infection is beneficial for infection.

### miRNA selection from bioinformatic analysis

A bioinformatic analysis of potential miRNAs targeting key lipid biosynthesis, transport, and cholesterol metabolism genes *abca1*, *abcg1*, *apoa1*, *apoe*, *apob*, and *ldlr* (Table 1) was performed. miRNA-mRNA pairs that appeared in at least two software predictions databases and were either experimentally observed or moderate-high predicted targeting were chosen to investigate their role in driving macrophage phenotypes and their interaction with MAP (Table 2).

**Table 1:** miRNA chosen for profiling in MAP infected macrophages.

miRNA	Potential lipid-associated function	Predicted targets
miR-129-5p (miR-129)	Foam cell formation	*Abcb1, Abcc5, Abcg1, Vldlr, Abcd2, Atg7*
miR-148a-3p (miR-148a)	Lipid uptake & foam cell formation	*Abca1, Abcb7*
miR-144-3p (miR-144)	Foam cell formation	*Abca1, Atg4, Vldlr, Abcg3, Ldlrad4*
miR-19a-3p (miR-19a)	Ox-LDL uptake & M2 macrophage phenotype	*Abca1, Abcb7, Abcg1*
miR-19b-3p (miR-19b)	Foam cell formation	*Abca1, Abcb7, Abcg1*
miR-24-3p (miR-24-3p)	Lipid accumulation & macrophage polarisation	*Insig1, Abcb9, Abcd1, Apob, Apoa5*
miR-24–2-5p (miR-24)	Lipid uptake	*Insig1, Apoa1, Abca1, Abcc9, Abcg8, Vldlr, Ldlrad4*
miR-425-5p (miR-425)	Lipid metabolism	*Abcc2, Abcf2, Apol1, Apoa1, Abcd1, Abca2*
miR-455-5p (miR-455)	M2 macrophage phenotype & cellular lipid metabolism	*Socs3, Scarb1, Ldlrad4*
miR-758-5p (miR-758)	Cholesterol efflux & M2 macrophage phenotype	*Abca1, Abcb7, Vldlr, Ldlrad2*

**Table 2:**
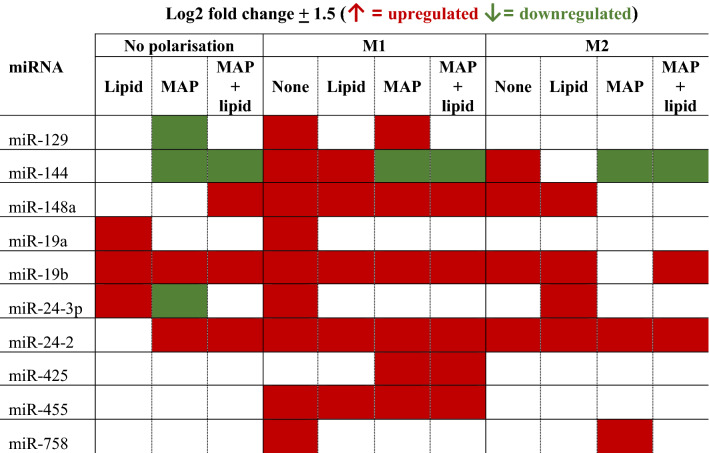
Change in miRNA expression in MAP infected murine macrophages.

Red boxes indicate upregulated miRNA and green boxes represent downregulated miRNA compared to control uninfected cells. While boxes indicate no significant differential expression. Log2 fold changes ± 1.5 compared to control uninfected cells was considered differentially regulated.

### Murine miRNA gene expression

**Figure 3:**
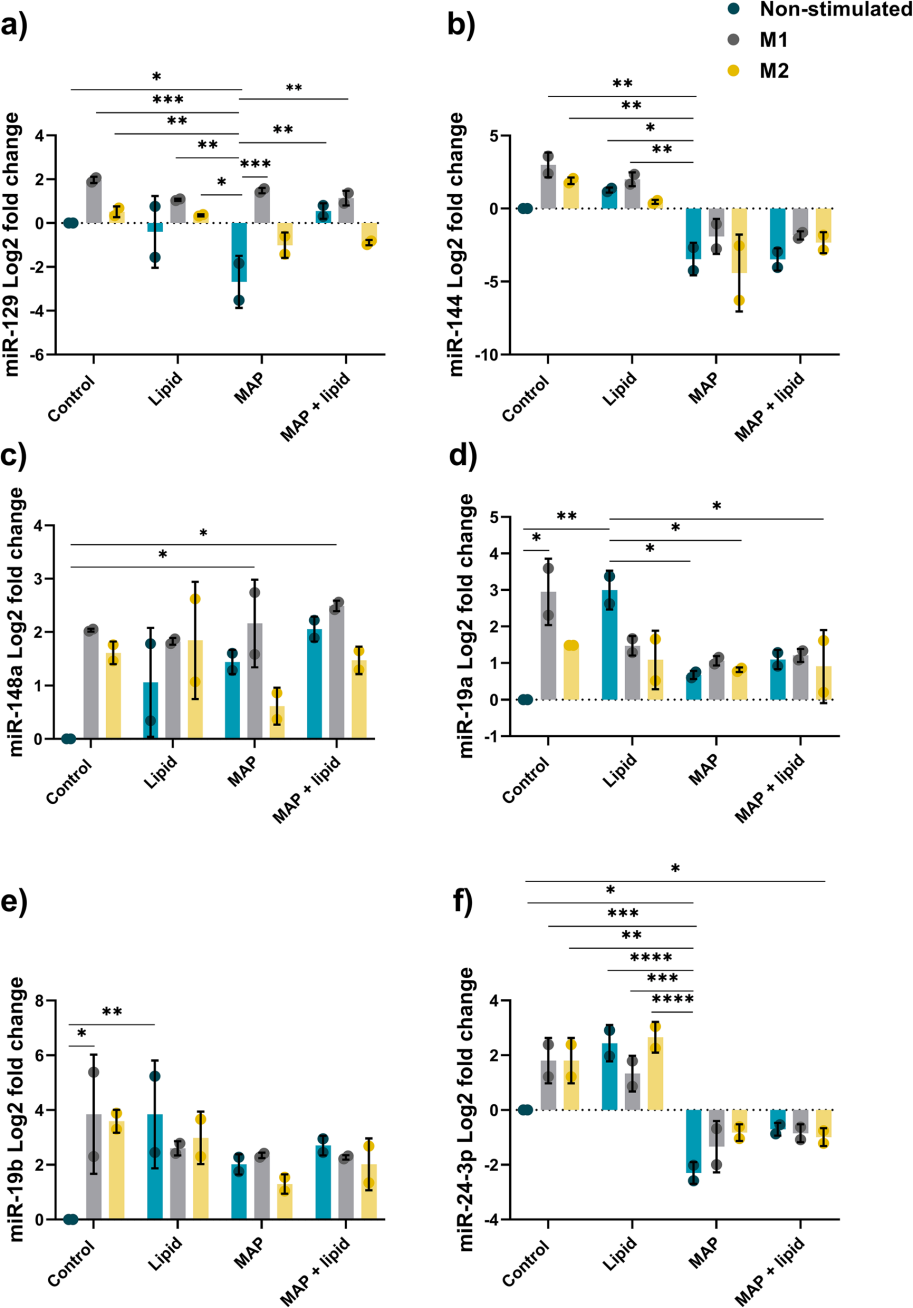
miRNA expression in MAP infected murine macrophages. RAW264.7 macrophages were infected with MAP with or without lipid supplementation. miRNA expression was analysed by qPCR at 24 hpi. (**a**) miR-129 fold change, (**b**) miR-24-3p fold change*,* (**c**) miR-144 fold change, (**d**) miR-24 fold change, (**e**) miR-19a fold change, (**f**) miR-148a fold change. Columns represent the mean fold change from control uninfected cells and error bars indicate the standard deviation, while asterisks indicate significantly different fold changes (*p ≤ 0.05, **p ≤ 0.01, ***p ≤ 0.001, ****p ≤ 0.0001). Graph is representative of 2 biological replicates and 2 technical replicates.

**Figure 4:**
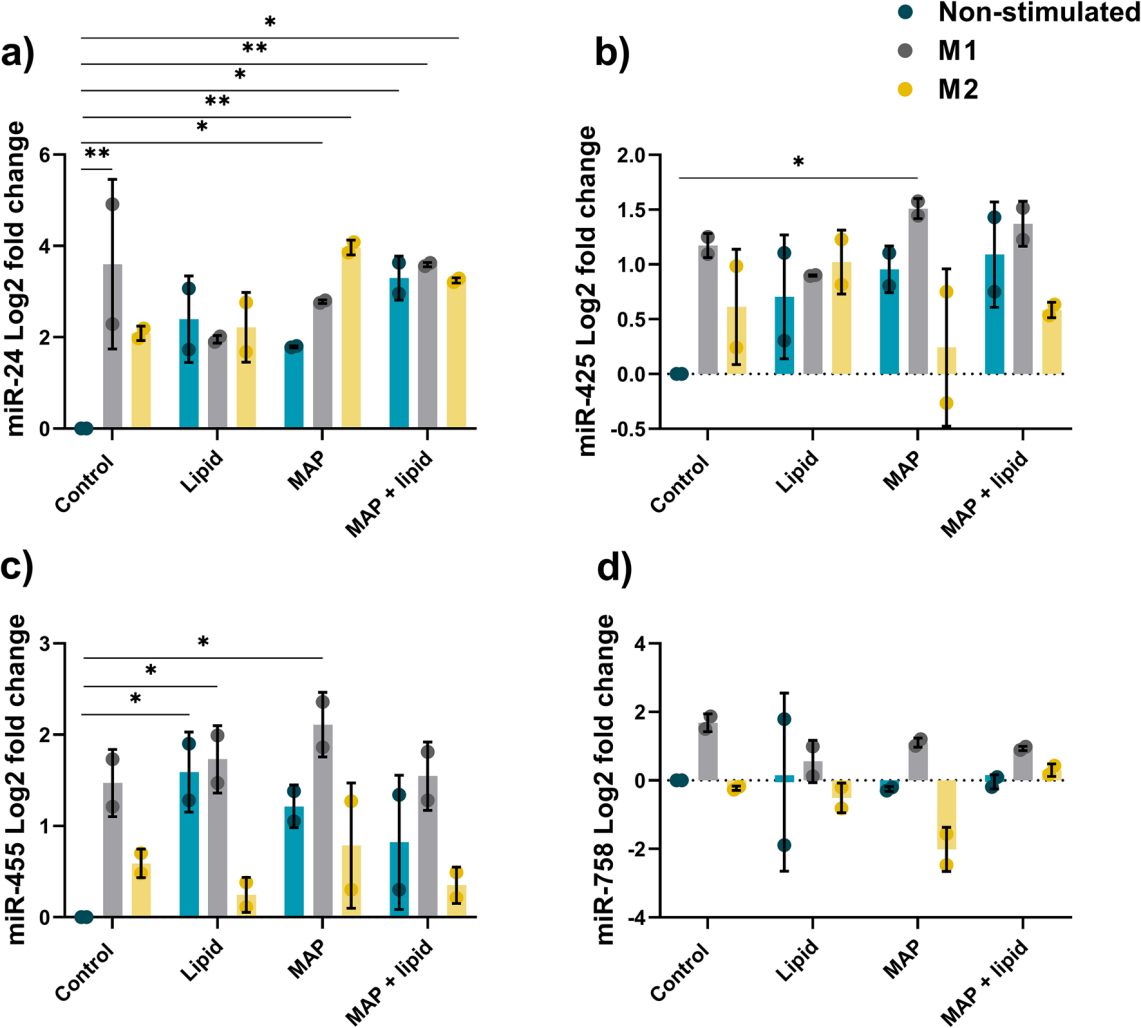
miRNA expression in MAP infected murine macrophages. RAW264.7 macrophages were infected with MAP with or without lipid supplementation. miRNA expression was analysed by qPCR at 24 hpi. (**a**) miR-19b fold change, (**b**) miR-455 fold change, (**c**) miR-425 fold change, (**d**) miR-758 fold change. Columns represent the mean fold change from control uninfected cells and error bars indicate the standard deviation, while asterisks indicate significantly different fold changes (*p ≤ 0.05, **p ≤ 0.01, ***p ≤ 0.001, ****p ≤ 0.0001). Graph is representative of 2 biological replicates and 2 technical replicates.

The effect of MAP infection and macrophage polarisation on miRNA expression was initially investigated using the murine macrophage cell line, RAW_264.7_. Ten miRNAs bioinformatically selected as having a role in lipid metabolism pathways and/or macrophage polarisation and activity were profiled at 24 hpi (Figs. 3, 4).

miR-129 expression was significantly decreased in MAP infected cells, however remained upregulated in all M1 polarised cells in comparison (Fig. 3a), potentially indicating the involvement of miR-129 in the response to MAP, that is further sensitive to M1 polarisation state and lipid supplementation. Similarly, miR-24-3p was significantly upregulated in all non-infected macrophages compared to MAP infected cells, regardless of polarisation phenotype or lipid supplementation (Fig. 3b). miR-24-3p was downregulated in all MAP infected macrophages, indicating a potentially MAP-driven response to suppress expression. Likewise, miR-144 was upregulated in all non-infected cells, except for M2 + lipid treatment, in comparison to MAP only cells (Fig. 3c). The addition of MAP to macrophages downregulated miR-144 in both M1 and M2 macrophages including those with lipid supplementation.

Expression of miR-24 was upregulated in M1 polarised uninfected macrophages and in all MAP infected cultures compared to control uninfected cells, with the exception of the control MAP infected cultures without lipid supplementation (Fig. 3d). This suggests that MAP, and MAP with access to excess lipid, may be driving expression of miR-24. However, there were no significant differences in miR-24 expression between the treatment groups (polarised, MAP-infected and/or lipid supplemented cultures). miR-19a showed similar expression patterns and was upregulated in M1 macrophages and lipid supplemented macrophages compared to both control uninfected macrophages, MAP infected, and M2 polarised infected and lipid supplemented macrophages (Fig. 3e). As lipid supplemented cells displayed an M1 phenotype with increased NO production (Fig. 1), miR-19a may be a regulator of lipid metabolism in MAP infection, with bacteria suppressing this response in infected and M2 macrophages.

While expression of miR-148a was upregulated in M1 polarised MAP infected cells, with or without lipid supplementation compared to control uninfected cells, there were no significant differences in expression between treatment groups (Fig. 3f), implying that miR-148a is associated with M1 inflammatory antimycobacterial macrophage responses. A similar M1 polarisation response was observed for expression of miR-19b, with upregulation in M1 and lipid supplemented cells compared to controls, however there were no significant differences found between the various treatment groups (Fig. 4a). miR-455 was upregulated in lipid supplemented macrophages with the exception of the M2 + lipid cultures, as well as in M1 polarised cells that were supplemented with either lipid or MAP, compared to the control uninfected cells (Fig. 4b). However, there was no significant regulation of this miRNA between polarised cells and those supplemented with MAP or lipid. Although miR-425 was upregulated in M1 infected macrophages compared to control uninfected cells, the expression between the treatment groups was not significantly different (Fig. 4c). Expression of miR-758 was not significantly regulated in macrophages following any treatment or supplementation (Fig. 4d).

Many of the miRNA were either strongly upregulated or downregulated in infected or lipid supplemented macrophages, as summarised in Table 2. Further analysis of their involvement in immune responses to infection may provide further understanding of the involvement of MAP in host lipid metabolism. Hence, miRs -19a, -24, -24-3p, and -129 were chosen as candidates for further investigation in bovine cells due to their modulation of expression by MAP.

### Bovine miRNA gene expression

Expression levels of four miRNA chosen for further investigation from murine studies was profiled in the BoMac cell line following MAP infection (Table 3). As the addition of MAP and/or lipid supplementation resulted in a primarily M1 inflammatory phenotype, regardless of previous polarisation status, we chose to assess miRNA expression only in MAP infected or control bovine macrophages.

**Table 3:**
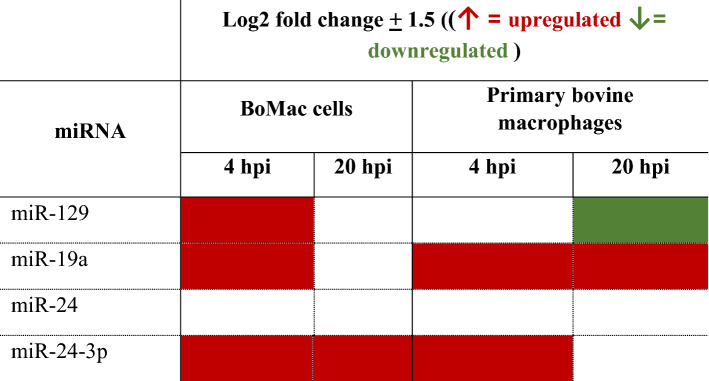
miRNA expression in MAP infected bovine macrophages.

Red boxes indicate upregulated miRNA and green boxes represent downregulated miRNA compared to control uninfected cells. While boxes indicate no significant differential expression. Log2 fold changes ± 1.5 compared to control uninfected cells was considered differentially regulated.

**Figure 5:**
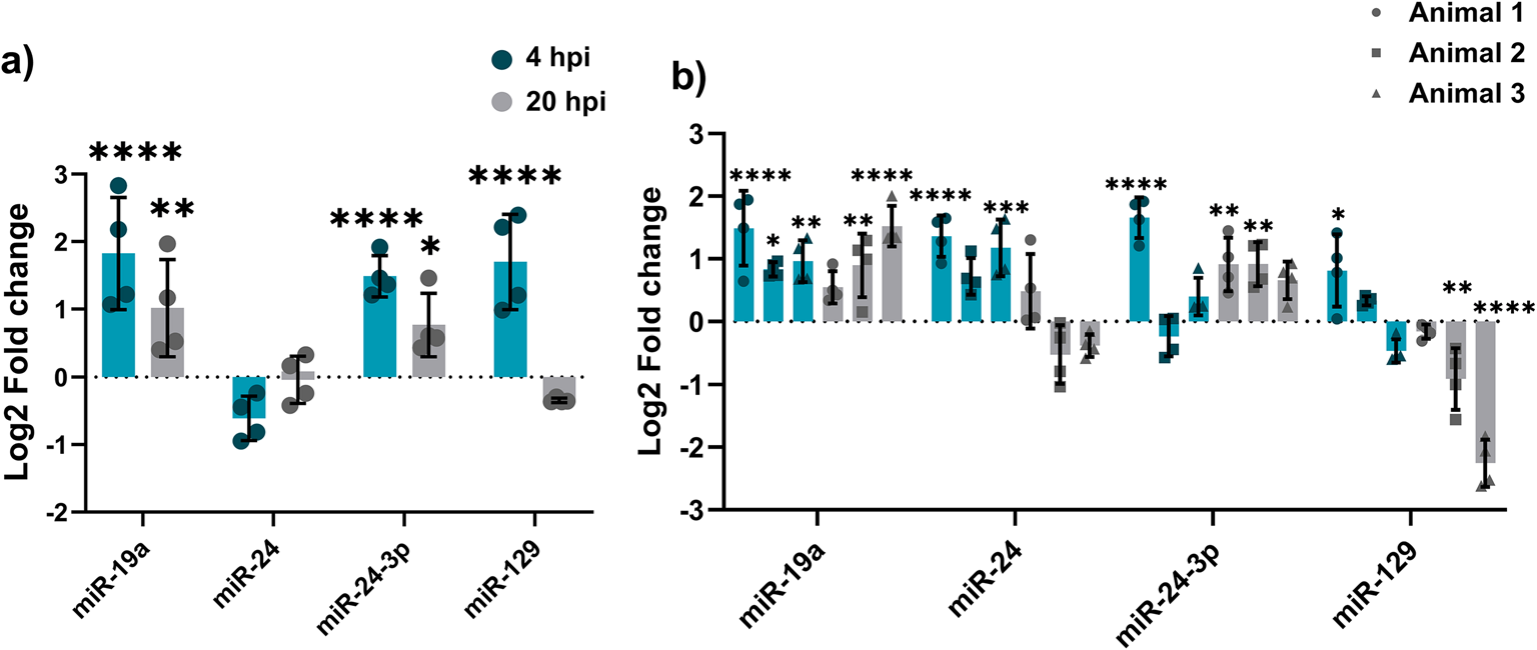
miRNA expression in MAP infected BoMac cells and primary bovine cells. (**a**) BoMac cells were infected with MAP and miRNA expression levels analysed by qPCR. (**b**) Primary bovine cells were cultured from PBMCs isolated from whole blood, infected with MAP, and miRNA expression analysed by qPCR. Columns represent the mean and error bars indicate the standard deviation. Asterisks indicate significantly different fold changes compared to control uninfected cells (*p ≤ 0.05, **p ≤ 0.01, ***p ≤ 0.001, ****p ≤ 0.0001). Graph is representative of 2 biological replicates for MAP infected cells and a single control uninfected sample, and 2 technical replicates of each experiment.

At 4 hpi, miRs -19a, -24-3p, and -129 were all significantly upregulated in infected cells compared to uninfected controls. At 20 hpi, miRs -19a and 24-3p remained increased, while expression of miR-129 was decreased. Despite a trend towards a decrease in transcript abundance of miR-24 at 4 hpi, there was no significant regulation at either timepoint (Fig. 5a).

**Figure 6:**
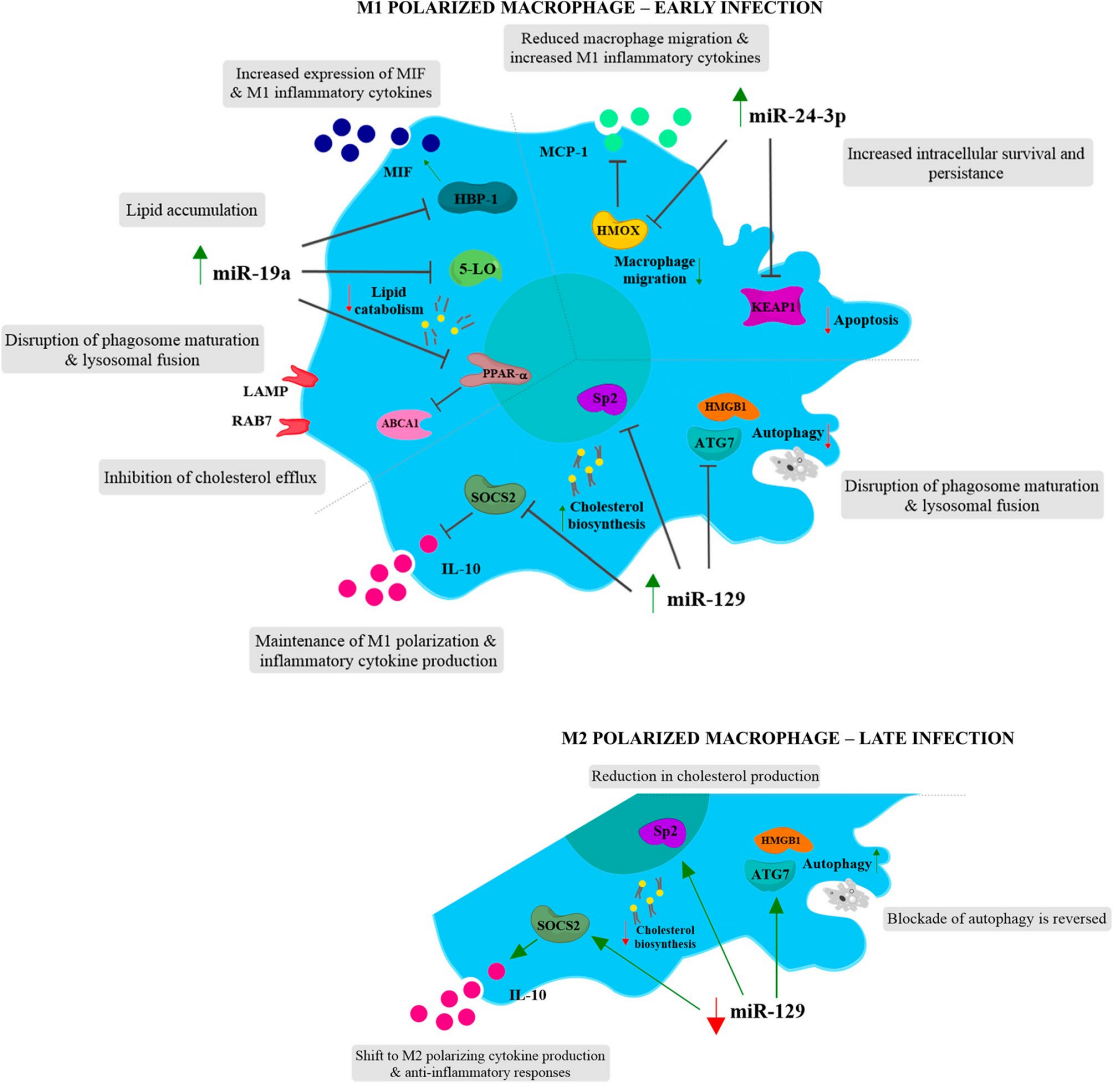
Summary of lipid-associated miRNA and their potential involvement in MAP infection. Regulation of miRNA by MAP can promote the formation of intracellular niches to prevent bacterial clearance. Through a decrease in targets 5-LO, HBP-1, and PPAR-α, increased miR-19a expression can promote foam cell formation through a decrease in lipid catabolism and M2 macrophage activation state. The interaction between PPAR-α and ABCA1, LAMP, and RAB7 further prevents lipid efflux and prevents phagosomal maturation in MAP infected macrophages. An increase in miR-129 in early MAP infection maintains an inflammatory M1 activation state through down regulation of SOCS2 and IL-10 production, as well as decreasing autophagy through HMGB1 and ATG7. In the later stages of infection, decreasing miR-129 increases IL-10 secretion and cholesterol biosynthesis through SP2, providing a source of energy for the bacteria. miR-24-3p controls macrophage polarisation state and migration through a HMOX/MCP-1 axis, along with augmenting apoptosis through target KEAP1.

To further confirm the relevance of expression profiles of these miRNA in primary cells, bovine macrophages were infected with MAP and miRs -24, -24-3p, -129a, and -19a quantified (Fig. 5b). In contrast to miRNA expression in MAP infected BoMac cells, primary bovine macrophages infected with MAP displayed a miRNA expression profile which was similar to the regulation in MAP infected murine macrophages. miR-19a and miR-24-3p were increased at both timepoints in infected primary bovine cells, whereas miR-24 and miR-129 displayed a switch from upregulated to downregulated from 4 to 20 hpi. Table 3 summarises miRNA expression in MAP infected BoMac cells and primary bovine macrophages, showing the modulation of transcription during the early stages of infection.

In MAP infected BoMac cells, similar expression patterns to murine RAW cell were observed for miR-19a and miR-129 at later timepoints (20–24 hpi), while miR-24 showed no regulation in either direction. Interestingly, miR-24-3p displayed opposing regulation in MAP infected in mouse and bovine cells, potentially due to variations in species-specific target binding and differing functional arms of pre-miRNA.

## Discussion

Macrophages are central to the phagocytosis and clearance of MAP by immune cells. However, they are also commandeered into providing an intracellular niche for the bacteria to persist and sustain infection^1^. Specific macrophage phenotypes direct the responses to invading pathogens. Broadly, the signals macrophages receive from their cellular milieu drive this polarisation. M1 polarised macrophages are pro-inflammatory and microbicidal, producing large amounts of TNF-α and NO. M2 macrophages are often anti-inflammatory and fail to kill mycobacteria^25,26^. It is therefore clear that polarisation of macrophages influences the outcome of infection at a cellular level and is likely to impact pathogenesis and disease outcome at the level of the whole animal^2^. In addition to host immune effectors, MAP itself can divert macrophage phenotype and therefore function to its benefit. Through modulation of miRNA transcript abundance, pathogenic mycobacteria are able to alter downstream target mRNA expression to moderate host immune responses^27^. miRNA provide another level of regulation through which mycobacteria can co-opt host signalling pathways to prevent clearance and establish infection^28–30^.

In this study, we aimed to investigate the effect of lipid supplementation on macrophage polarisation and miRNA expression to reveal MAP driven responses to regulate host lipid metabolism. The role of lipids in macrophage polarisation is of particular interest, as MAP and mycobacteria actively utilise host lipid pathways to aid persistence and block host defence mechanisms^14–17,31^. Lipogenesis and fatty acid synthesis are key processes in inflammatory immune responses, driving macrophages towards an M1 phenotype and activating microbicidal inflammasomes^32–34^. Conversely, lipolysis is believed to be a driver of M2 macrophage functions, perpetuating an anti-inflammatory response^35^. The interaction between these host processes and bacterial survival strategies may alter immune responses to MAP.

We found that lipid supplementation skewed macrophage phenotypes towards an M1 state. As lipid supplementation resulted in foam cell formation and reduced bacterial clearance in M1 polarised and supplemented macrophages, we concluded that inflammatory macrophages and utilisation of host lipids were key pathways regulated by MAP following infection. Further investigation of post-transcriptional regulation of these pathways provided four miRNA which appear to be responsive to infection and associated with lipid related functions.

Previous studies have associated miR-129 with macrophage polarisation and control of mycobacteria through targeting of SOCS2 and the Sp2 transcription factor^36–38^. Decreased miR-129 transcripts at 20–24 hpi in MAP infected macrophages may promote eventual M2 polarisation through increased SOCS2 and Sp2 target expression, responsible for IL-10 induction. During early infection (4 hpi), miR-129 was upregulated, likely maintaining the M1 phenotype, before switching to a pro-survival M2 phenotype in the later stages of early pathogenesis (20–24 hpi). Further, miR-129 has been shown to regulate autophagy through ATG7 and HMGB1, potentially facilitating disruption of the phagolysosomal pathway by MAP^39,40^. Supplementation of macrophages with exogenous lipid rescued the M1 polarisation phenotype in MAP infected cells, indicating a lipid dependant function of miR-129. A potential target of miR-129, transcription factor Sp2, is known to regulate cholesterol and lipid biosynthetic pathways^41^, and may explain the increased bacterial clearance observed in M1 polarised lipid rich macrophages.

miR-24-3p elicited a similar response to lipid supplementation and MAP infection in murine macrophages but not bovine macrophages. In murine macrophages infected with MAP, miR-24-3p expression was reduced compared to non-infected cells, regardless of polarisation state or lipid supplementation. While this infection-driven reduction in miR-24-3p transcription was not apparent in MAP infected bovine macrophages, there was a trend towards reduced expression from early to later timepoints, however, may represent unrelated temporal changes in expression rather than MAP-dependant changes. miR-24 is highly conserved between species, and while both miR-24 and miR-24-3p arise from the same pre-miRNA molecule, they may possess different functional capabilities across species. Expression of miR-24 in bovine cells was similar to that of miR-24-3p, suggesting that they may share functions and bioactivity rather than being independently regulated as observed in murine infections.

As miR-24-3p contributes to the attenuation of phagocytosis and promotes alternative or M2 macrophage activation^42–44^, increased miR-24-3p following MAP infection may promote an M2 macrophage state, suppressing the MAP-associated inflammatory phenotype. Further suggested roles for miR-24-3p include interference with antigen presentation in myeloid cells and the regulation of apoptotic pathways, further supporting the apparent mechanism of miR-24-3p in suppressing the infection-associated M1 inflammatory phenotype in host macrophages following MAP infection^45–47^. Further, miR-24-3p has been shown to suppress heme oxygenase (*hmox*), which has recently been implicated in macrophage migration in mycobacterial infection, suggesting that altered miR-24-3p expression may be impacting macrophage activation and activity^48,49^.

Another miRNA of interest from our work is miR-19a, an apparent M1 inflammatory marker. In murine macrophages, miR-19a was strongly upregulated in M1 and lipid supplemented cells while the magnitude of this increase was dampened following infection with MAP. An increase in MAP infected macrophages may contribute to the observed foam cell formation and reduction in cellular lipid efflux. miR-19a acts on several lipid metabolism pathways to regulate lipid efflux and mediate inflammation, while reduced expression in MAP infected macrophages suggests this infection-responsive miRNA may be modulated by MAP to promote survival. Through a reduction in its targets 5-lipoxygenase, HBP-1, and PPAR-α, miR-19a is able to reduce lipid efflux and catabolism, promoting foam cell formation and providing potential metabolic fuel to intracellular MAP^36,50–53^.

The interaction between MAP and macrophages is paramount to host control and disease progression, and the complex relationship between mycobacteria and host lipids further complicate immune responses. Infection with MAP led to an M1 inflammatory response, suggesting that during early pathogenesis MAP may utilise lipid pathways to support the M1 macrophage state and in turn, their persistence within cells. Changes in miRNA expression throughout the progression of early infection further suggests that MAP is able to manage host miRNA responses to change the cellular microenvironment as infection progresses.

The ability of MAP and virulent mycobacteria to modulate gene expression and host lipid metabolism pathways for their survival is a multifaceted process; however, we have shown that there is a further level of regulation by non-coding miRNA. This provides MAP with another means to control host gene expression and alter effective immune function. As summarised in Fig. 6, MAP infection is able to alter host miRNA expression to affect downstream targets; however, the pleiotropic nature of miRNA and their multitude of targets makes interpretation of effector pathways difficult. This further highlights the need for context-dependant functional studies as miRs may exhibit differing regulation under different biological conditions.

**Figure 7:**
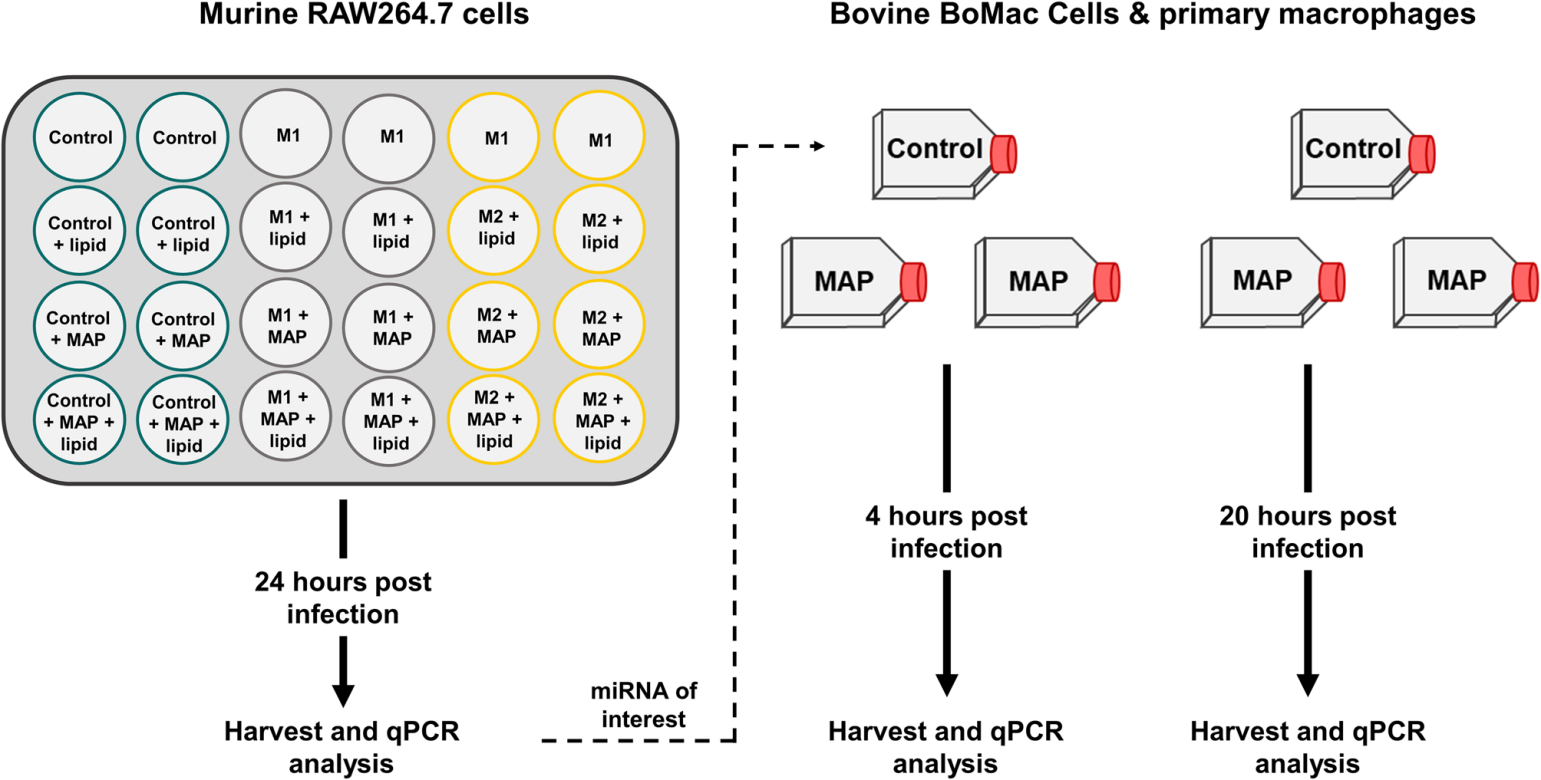
Experimental overview. Graphical illustration of cultured murine and bovine cells. Murine RAW264.7 cells were polarised to an M1 or M2 phenotype and further infected with MAP and/or supplemented with lipid. miRNAs of interest were then analysed in control uninfected or MAP-infected BoMac cells and primary bovine macrophages.

This study utilised several in vitro models to uncover MAP-driven miRNA dysregulation. While murine RAW264.7 macrophages and primary bovine macrophages displayed similar expression profiles, there was a discrepancy between BoMac cells and primary bovine macrophages. Although disparities in the magnitude of expression and directionality between murine and bovine cells can be partially explained by species-specific miRNA roles; the differential expression between BoMac and primary macrophages suggests activation of alternate phagocytic pathways. BoMac cells lack cell surface CD14 receptors, several integrin receptors and have lower phagocytic capacity for MAP as well as a reduced ability to allow its intracellular replication^54,55^. The combination of these factors may explain the differences in induction or inhibition of miRNA, with the interaction of MAP and BoMac cells activating non-CD14/TLR dependant phagocytosis and engulfment, which leads to alternate signalling pathways and intracellular conditions^55,56^. The use of these multiple in vitro models of MAP infection provides further insight into the cellular cues which drive miRNA expression during infection. These results provide an interesting starting point for the investigation of key pathogen recognition and signalling molecules involved in MAP-dependant miRNA expression.

Analysis of infection-induced gene expression has further contributed to the growing knowledge of miRNA control of host immune responses, and their alteration following mycobacterial infection. While we selected only a few miRNAs to profile, further studies to uncover lipid-associated miRs may provide key molecules and pathways altered during infection and provide potential therapeutics for the treatment of mycobacterial infection. To conclude, we have shown that miRs -129, -24-3p, and -19a are responsive to macrophage polarisation and that MAP is able to alter their expression to exploit lipid metabolism and macrophage polarisation pathways.

## Methods

### miRNA target prediction and bioinformatics

To assess the role of miRNA in macrophage polarisation and lipid metabolism during MAP infection, key genes involved in these pathways were chosen to investigate their regulation by miRNA. Previous work has identified lipid biosynthesis and transport genes *abca1, apoe,* and *ldlr* as being deferentially regulated during infection and contributing to cholesterol accumulation in macrophages^14^. These genes were therefore chosen, along with related lipid transporters genes *abcg1, apoa1,* and *apob*, as genes of interest for miRNA targeting prediction. To identify miRNA that target these genes of interest 4 online databases (Ingenuity Pathway Analysis, microT-CDS 5.0, miRWalk 2.0, TargetScan 7.2) were queried for miRNA-mRNA interactions. Potential lipid-associated miRNAs were selected if they appeared in at least 2 databases and showed experimentally observed or moderate-high predicted targeting of the genes of interest.

### Preparation of bacterial suspension

Frozen bacterial suspensions (S strain Telford 9.2; C strain C0912), prepared as previously described^57^, were thawed and washed with PBS. Antibiotic-free culture media was added up to 1 mL and the mixture passed through a fine gauge needle to ensure a single cell suspension. MAP suspensions were stored at 4 °C until required and used within a week at an MOI of 10.

### Cell culture and macrophage polarisation

Cell culture treatments and workflow are summarised in Fig. 7.

#### RAW264.7 cells

Murine macrophages (RAW 264.7) (European Collection of Cell Cultures) were cultured in 2 duplicate 24-well flat bottom culture plates (Greiner Bio-One, Australia) in complete culture medium (Dulbecco’s Modified Eagle’s Medium) (DMEM), (Gibco, USA) containing 10% foetal calf serum, (Gibco, USA), 1% 100 U/mL penicillin/100 µg/mL streptomycin (Gibco, USA) and 0.1% 29 mM β-mercaptoethanol (Sigma-Aldrich, USA). All incubations were carried out in complete culture medium at 37 °C and 5% CO_2_. Cells were seeded at 2.5 × 10^5^ cells per well and incubated for 24 h prior to inducing polarisation with either 25 ng/mL *E. coli* LPS and 20 ng/mL IFN-γ (M1), or 20 ng/mL IL-4 (M2). Briefly, media was removed from each well and replaced with 500 µL media containing either M1 cytokines or M2 cytokines, or media only for control unstimulated samples. Following a 6-h incubation, polarised cells were further subjected to lipid supplementation or MAP infection. Macrophage Serum-Free Media (MSFM) (Gibco, USA) was used to minimise lipids in the culture medium. MSFM was added to control wells, and other wells supplemented with either lipid (25 µL/mL of media) (Sigma-Aldrich, USA) or MAP (S strain Telford 9.2) at an MOI of 10:1 or both lipid and MAP. Culture plates were incubated for 24 h prior to harvesting. Macrophage phenotype was confirmed by qPCR analysis of M1 marker (*nos2*) and M2 marker (*arg-1*) and cellular NO production (Griess assay).

#### BoMac cells

Bovine macrophages (BoMac) were cultured in duplicated T25 cell culture flasks (Greiner Bio-One, Australia) at a density of 7.5 × 10^5^ cells in 5 mL of RPMI complete culture media and incubated under standard conditions. Cells were rested for 24 h to allow for adherence prior to infection. Media from all flasks was removed and replaced with 5 mL of antibiotic-free media. MAP (C strain C0912) was added to each of the infected flasks at MOI 10:1 and all flasks incubated for the required time.

#### Primary bovine cells

Peripheral blood mononuclear cells (PBMC) were isolated by density gradient centrifugation and layering over Ficoll-Paque Plus (GE Healthcare, Australia) as previously described^58^. Briefly, blood was collected from the jugular vein of 3 healthy cattle into lithium heparin vacutainer tubes and centrifuged. Buffy coats were then layered over Ficoll-Paque Plus and white blood cells collected and resuspended in DMEM complete culture media. PBMCs were maintained in DMEM with 20% FBS and stimulated with rHuM-CSF (1 ng/mL) (Sigma-Aldrich, USA) to differentiate adhered PBMCs into monocyte-derived macrophages (MDM), identified by visual phenotyping of cytoplasmic granules, elongated shape, and characteristic cellular protrusions. Adhered cell0073 were cultured in duplicate in DMEM with 20% FBS without antibiotics and infected with MAP (C strain C0912) at MOI 10:1 and incubated for the required time.

### Cell harvesting and RNA extraction

For the harvest of RAW 264.7 cells, culture supernatant was aspirated and stored at − 20 °C for future use, and 500 µL of RNAzol RT (Sigma-Aldrich, USA) was added to the well and mixed before transferring the entire volume to a 1.5 mL screw cap tube on ice. For BoMac and primary macrophages, media was removed, and flasks were washed with 1 mL of RNAzol RT (Sigma-Aldrich, Australia), and the lysate transferred to a 2 mL tube and frozen at − 80 °C until processed. RNA was extracted from RNAzol according to the manufacturers protocol. RNA was resuspended in 50 µL of nuclease-free water and quantity and purity measurements performed on a NanoDrop 1000 spectrophotometer measuring absorbance at 260 nm.

### DNase treatment

Genomic DNA was removed using the RQ1 RNase-Free DNase kit (Promega, USA) according to the manufacturer’s instructions. A standard input of 2.5 µg was added to each reaction along with 5 µL of RQ1 10 × Reaction Buffer, 5 µL RQ1 DNase enzyme, 1 µL of RNasin Plus RNase Inhibitor, and nuclease-free water up to a total volume of 50 µL. Following DNase treatment, RNA samples were purified in 0.1 volumes of 3 M sodium acetate (pH 5.5) and 2.5 volumes of 100% molecular grade ethanol and incubated overnight at − 20 °C. Cleaned RNA was then resuspended in 30 µL of nuclease-free water.

### cDNA synthesis

For mRNA expression, cDNA was synthesised using the SensiFAST cDNA Synthesis Kit according to the manufacturer’s instructions. A standard volume of 15 μL of RNA was used reverse transcription reactions and cDNA diluted to 1/100 prior to use in qPCR.

cDNA synthesis for miRNA expression profiling was carried out using the miScript Reverse Transcription Kit (Qiagen, Germany) according to the manufacturer’s instructions. For each reaction, 100 ng of RNA was transcribed to cDNA and diluted 1/100 for use in qPCR reactions.

### qPCR

mRNA qPCRs were performed using Quantitect SYBR Green PCR Mastermix and primer concentration of 300 nM. miRNA qPCRs were performed using the miScript SYBR Green PCR Kit (Qiagen, Germany) consisting of Quantitect SYBR Green PCR Mastermix, with the miScript Universal Primer used alongside miR specific miScript primer assay. All qPCRs were carried out on an Mx3000p Real-time PCR system.

10 murine miRNAs (miR-148a-3p, miR-144-3p, miR-19a-3p, miR-19b-3p, miR-24-3p, miR-24–2-5p, miR-758-5p, miR-129-5p, miR-455-5p, miR-425-5p) and 5 bovine miRNAs (bta-miR-24-3p, bta-miR-24, bta-miR-19a, bta-miR-129, bta-miR-148-3p) were assessed. Cross-species conservation was checked by comparing mature miRNA sequences on miRBase, and mouse primers were used for bovine miRs -129-5p, -19a-3p, and -24-3p. miRNA primers were obtained from Qiagen (Table 4) and were resuspended in 550 µL of TE buffer (pH 8.0) prior to use.

**Table 4:** List of primers used in this study.

Primer	Target sequence 5’-3’	GeneGlobe ID
mmu-miR-129-5p	CUUUUUGCGGUCUGGGCUUGC	MS00006020
mmu-miR-144-3p	UACAGUAUAGAUGAUGUACU	MS00032326
mmu-miR-148a-3p	UCAGUGCACUACAGAACUUUGU	MS00001652
mmu-miR-19a-3p	UGUGCAAAUCUAUGCAAAACUGA	MS00001302
mmu-miR-19b-3p	UGUGCAAAUCCAUGCAAAACUGA	MS00005915
mmu-miR-24-3p	UGGCUCAGUUCAGCAGGAACAG	MS00005922
mmu-miR-24-2-5p	GUGCCUACUGAGCUGAAACAGU	MS00011550
mmu-miR-425-5p	AAUGACACGAUCACUCCCGUUGA	MS00012012
mmu-miR-455-5p	UAUGUGCCUUUGGACUACAUCG	MS00006321
mmu-miR-758-5p	UGGUUGACCAGAGAGCACACG	MS00026397
bta-miR-24	GUGCCUACUGAGCUGAUAUCAGU	MS00052458
RNU6		MS00033740
β-actin	qFw- GGCTATGCTCTCCCTCACG qRv- CACGCTCGGTCAGGATCTT	

A standard curve was included for each miRNA on each plate using a fivefold dilution of neat cDNA. Amplicon specificity was confirmed with a dissociation curve and the standard curve analysed to ensure efficiency between 90–110%, slope between 3.1–3.6, and R^2^ > 0.98. Data was normalised to either β-actin or U6 and analysed using the 2^-ΔΔ^ Ct method.

### Griess test

A Griess assay was performed to assess nitric oxide secretion from polarised cells. The Griess test measures nitrite within cell culture supernatant to evaluate the NO production. Briefly, 1 × Griess Reagent (modified) (Sigma-Aldrich, USA) was prepared by adding 250 mL of ultrapure Milli-Q water to the bottle and mixed by inverting. A standard curve was generated for each plate using a serial dilution of 97% sodium nitrite (Sigma-Aldrich, USA) and serum-free culture media. Culture supernatant (50 µL) from each sample (in duplicate) mixed with 50 µL of 1 × Griess Reagent. Plates were covered from light and gently mixed for 2 min using a plate shaker and incubated in the dark for 50 min. Absorbance at 540 nm was measured using a plate reader.

### Statistical analysis

Statistical analysis was performed in GraphPad Prism (v.9.0.0) using default parameters. For qPCR analysis, raw Cq values were normalised to housekeepers U6 pseudogene (miRNA analysis) or β-actin (mRNA analysis) and analysed using the 2 ^−ΔΔ^ Ct method^59^. Genes were considered differentially expressed if there was a statistically significant difference in the fold change compared to control. P values were calculated using a one-way ANOVA followed by Tukey’s post-hoc analysis to determine differences between pairwise comparisons, with significance at the 5% level. P values are represented by asterisk’s: *p ≤ 0.05, **p ≤ 0.01, ***p ≤ 0.001, ****p ≤ 0.0001.

## Supplementary Information

Supplementary Information.

## Data Availability

Analysed data are presented within the current manuscript. Individual raw data is available from the corresponding author upon reasonable request.
